# International Consensus Recommendations for the Assessment and Management of Individuals With CDKL5 Deficiency Disorder

**DOI:** 10.3389/fneur.2022.874695

**Published:** 2022-06-20

**Authors:** Sam Amin, Marie Monaghan, Angel Aledo-Serrano, Nadia Bahi-Buisson, Richard F. Chin, Angus J. Clarke, J. Helen Cross, Scott Demarest, Orrin Devinsky, Jenny Downs, Elia M. Pestana Knight, Heather Olson, Carol-Anne Partridge, Graham Stuart, Marina Trivisano, Sameer Zuberi, Tim A. Benke

**Affiliations:** ^1^Department of Paediatric Neurology, Bristol Royal Hospital for Children, Bristol, United Kingdom; ^2^Epilepsy Program, Department of Neurology, Ruber Internacional Hospital, Madrid, Spain; ^3^Pediatric Neurology, Necker Enfants Malades, Université de Paris, Paris, France; ^4^Royal Hospital for Sick Children, University of Edinburgh, Edinburgh, United Kingdom; ^5^University Hospital of Wales, Cardiff University, Cardiff, United Kingdom; ^6^Developmental Neurosciences, UCL NIHR BRC Great Ormond Street Institute of Child Health, London, United Kingdom; ^7^Departments of Pediatrics and Neurology, University of Colorado School of Medicine, Children's Hospital Colorado, Aurora, CO, United States; ^8^Department of Neurology, New York University, New York, NY, United States; ^9^Telethon Kids Institute, The University of Western Australia, Perth, WA, Australia; ^10^School of Physiotherapy and Exercise Science, Curtin University, Perth, WA, Australia; ^11^Cleveland Clinic Epilepsy Center, Cleveland Clinic Learner College of Medicine, Cleveland, OH, United States; ^12^Division of Epilepsy and Clinical Neurophysiology, Department of Neurology, Boston Children's Hospital, Boston, MA, United States; ^13^CDKL5 UK, Somerset, United Kingdom; ^14^Bristol Heart Institute, Bristol Royal Hospital for Children, University of Bristol, Bristol, United Kingdom; ^15^Rare and Complex Epilepsy Unit, Department of Neuroscience, Bambino Gesù Children's Hospital, IRCCS, Rome, Italy; ^16^Paediatric Neurosciences Research Group, Royal Hospital for Children, Glasgow, United Kingdom; ^17^College of Medical, Veterinary and Life Sciences, University of Glasgow, United Kingdom; ^18^Department of Pediatrics, Pharmacology, Neurology, and Otolaryngology, University of Colorado School of Medicine, Children's Hospital Colorado, Aurora, CO, United States

**Keywords:** CDKL5 deficiency disorder, cyclin-dependent kinase-like 5, developmental and epileptic encephalopathy, care guideline, consensus methods, Delphi methods

## Abstract

CDKL5 Deficiency Disorder (CDD) is a rare, X-linked dominant condition that causes a developmental and epileptic encephalopathy (DEE). The incidence is between ~ 1:40,000 and 1:60,000 live births. Pathogenic variants in *CDKL5* lead to seizures from infancy and severe neurodevelopmental delay. During infancy and childhood, individuals with CDD suffer impairments affecting cognitive, motor, visual, sleep, gastrointestinal and other functions. Here we present the recommendations of international healthcare professionals, experienced in CDD management, to address the multisystem and holistic needs of these individuals. Using a Delphi method, an anonymous survey was administered electronically to an international and multidisciplinary panel of expert clinicians and researchers. To provide summary recommendations, consensus was set, *a priori*, as >70% agreement for responses. In the absence of large, population-based studies to provide definitive evidence for treatment, we propose recommendations for clinical management, influenced by this proposed threshold for consensus. We believe these recommendations will help standardize, guide and improve the medical care received by individuals with CDD.

## Introduction

CDKL5 deficiency disorder (CDD) is a rare and X-linked dominant condition (1, 2), with many aliases, including Developmental Epileptic Encephalopathy 2 (DEE2) (3, 4). It is caused by loss-of-function variants in the *CDKL5* gene (5) which maps to Xp22.13, a gene with 20 coding exons (6, 7). The gene codes for Cyclin-Dependent Kinase-like 5 (CDKL5) protein, previously known as Serine-Threonine Kinase 9 (STK9) (8). *CDKL5* was first mapped by Montini et al. (9) before subsequently seeing an update to its described genomic structure in 2003 (6) by Kalscheuer and colleagues. It was at this time that *CDKL5* was reported as the second cause of X-linked infantile spasms (ISSX), for the first time highlighting genetic heterogeneity in this clinical syndrome. Further genetic reports followed, describing *CDKL5* variants as disease causing while also being genetically and clinically distinct from Rett syndrome (10–12).

As an X-linked dominant condition, CDD is more frequently found in females, with a varying report of female-to-male ratio of between 4:1 (2) up to 12:1 (13). Males are described as displaying a more severe phenotype. The incidence is estimated at between ~ 1:40,000 and 1:60,000 live births, approximating to one-third of the frequency of Dravet syndrome (1:20,000–1:50,000) (14, 15) or one-quarter of the frequency of Rett syndrome (1:10,000) (16). It is detected in 10–20% of females with early-onset DEEs presenting within the first 6 months of life and should be considered as part of a differential diagnosis for children, females and males, presenting with severe, early-onset epilepsy (17).

CDD presents with a broad phenotype that includes intellectual disability, and impairments in speech, gross and fine motor abilities (18), sleep, gastrointestinal function (19) and vision. Approximately 75% of individuals have cortical visual impairment (20, 21). Seizures typically present in early infancy, with a wide spectrum of semiologies, and are often refractory to treatment (22, 23). Criteria for recognition and diagnosis have been proposed to guide clinicians (2). Evidence is emerging of genotype phenotype correlations for CDD gene variants (24). Evidence-based guidelines have recently been suggested for another DEE, Rett syndrome (25) but there is currently a paucity of evidence and no published consensus to guide clinical management in CDD. Given the broad phenotype, unique features and rarity of CDD, an initial document describing comprehensive care is needed to assist specialist and primary care practitioners caring for individuals diagnosed with CDD. Accordingly, we reviewed the literature and used consensus methods to establish recommendations for clinical management in CDD.

## Methods

*Study design:* Delphi method.

*Literature review and initial guideline development:* We performed a literature search and considered mortality, morbidities, diagnosis, treatment, and surveillance of CDD. We used Medline/Pubmed and the Cochrane Library to perform the search.

The main search terms were: “CDKL5” and “Cyclin-dependent Kinase-Like 5.” Other associated search terms, as relevant to the topics of interest, were also searched for in combination with the main search terms.

**Figure 1:**
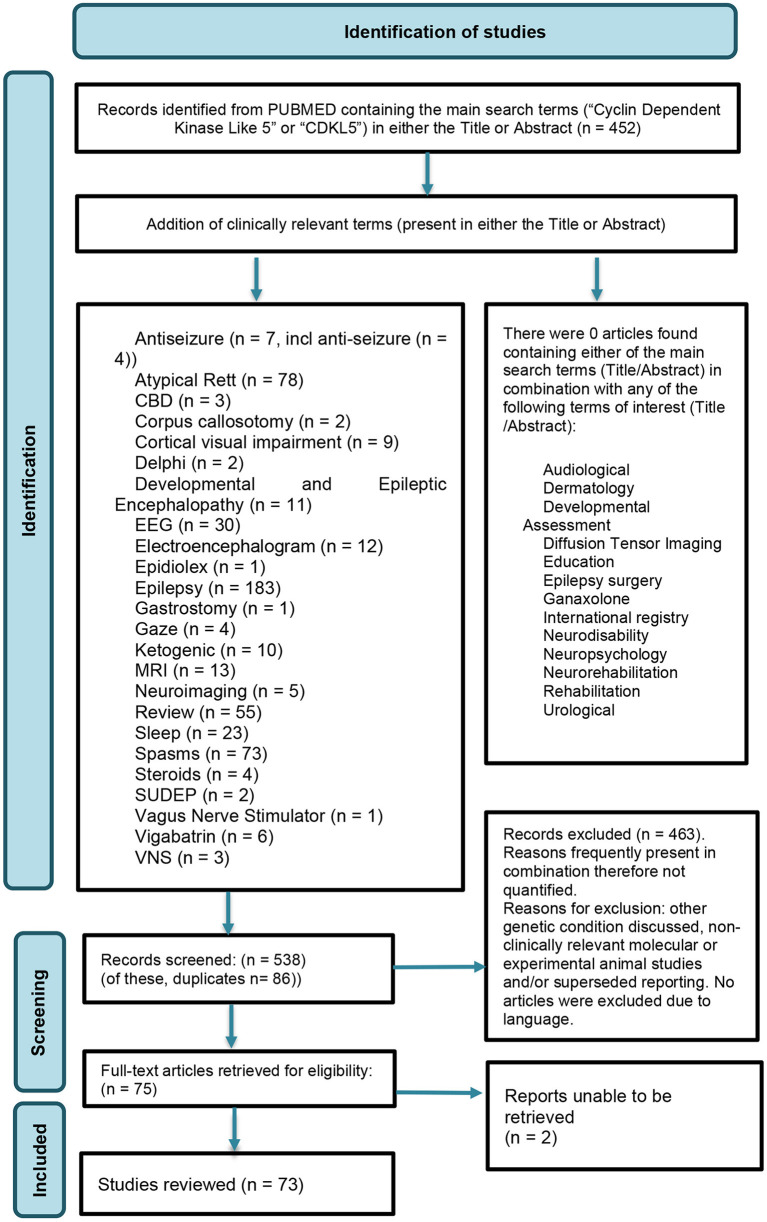
Identification of studies.

Searching of the Cochrane Library (26) yielded no articles featuring either of the two main search terms. Searching Medline (Pubmed) (27) for the presence of either of the two main search terms, within title or abstract, yielded 452 articles. The search term, “CDD” was not used, to avoid unrelated terms or conditions with the shared abbreviation e.g., “cervical degenerative disease” (CDD). To inform the questions of the survey, the evidence on each topic was reviewed by filtering the main search term results (*n* = 452) to identify topics of interest as included in the title or abstract (Figure 1).

Based on these findings, we identified 84 questions for consideration in the Delphi process. The items queried all aspects of CDD including initial assessments, diagnosis, treatment options, follow-up, and surveillance.

The questions were formed by a core committee and reviewed by a subcommittee. Families and caregivers also contributed by review of the design. The Delphi results were analyzed by committees, which consisted of experts in different aspects of CDD from the US, Europe, and the UK. Patient advocacy groups were also part of this process.

*Delphi consensus method:* The Delphi process provides consensus guidance for the delivery of best clinical care. It is important that the participants are selected carefully. One potential pitfall in a consensus process is that when questions address issues without an evidence base, some respondents may provide answers despite a lack of specific knowledge. It is crucial, therefore, that the respondents are experts in the field. It is generally believed that 15–20 subjects could be sufficient to take part in a Delphi process but the higher the number of the subjects and homogeneity of response, the better the outcome. Some papers have cited that consensus thresholds can be accepted even low as 51%, “in keeping with most other Delphi studies” (28) with others recommending that consensus thresholds should be higher or require unanimous agreement, depending on the gravity of the decisions being made (29). Reviews of Delphi methodology describe the varying nature of the consensus thresholds but all note the importance of having a pre-defined threshold level for consensus, to avoid author bias upon review of responses (30). For this project, *a priori* consensus was defined as 70% agreement for all areas.

General pediatricians, pediatric neurologists, ophthalmologists, developmental pediatricians, geneticists, orthopedic surgeons, adult neurologists, rehabilitation clinicians, allied health professionals, gastroenterologists and nutritionists were invited to take part. All the people surveyed were based in the US, Europe, and the UK. Clinicians were identified through CDD clinics and Centers of Excellence, and researchers were identified through publications. The surveys were conducted over 6 months from August 2020 to January 2021. A weekly electronic reminder was sent to the responders. Forty-seven experts responded to the survey. The responders were pediatric neurologists (*n* = 30), epileptologists (*n* = 10), geneticists (*n* = 2) as well as a general pediatrician, a development/community pediatrician and an allied health professional. Two of the 47 respondents did not describe their specialty. The number of years of experience within their current specialty favored highly experienced professionals, with 58% (*n*= 27) having >15 years, followed by 34% (*n* = 16) with >5 years of experience. Professionals had a mixed range of experience in managing CDD; under half had managed <5 individuals (*n* = 22, 47.8%) followed by nearly a fifth that had managed 6–10 individuals (*n* = 9, 19.6%) with nearly a third (32%) having managed >10 individuals with CDD (*n* = 15). While CDD exposure had been mixed, most of the surveyed professionals (*n* = 46, 97.9%) had significant (>20 individuals) experience in managing DEEs. Many members of the core and subcommittee had a wealth of expertise in managing patients with CDD, leading their national centers of excellence in their practicing countries.

## Results

The survey contained questions relating to current practice in CDD and was sent to the respondents. Answers where respondents did not feel they had relevant experience to be able to answer, indicated by selection of “I am not qualified to answer” or “I do not know,” were excluded, for the purposes of assessing the degree of consensus of opinion among experienced responders for each particular topic. Questions referring to “at baseline” were in reference to where the diagnosis of CDD had already been made, with the exception of genetic testing (CDD is considered a genetic diagnosis).

### Genetic Screening and Counseling

ACMG (American College of Medical Genetics and Genomics) guidelines state that genetic counseling should be “offered at all stages of genetic testing”.

#### Survey

There was consensus in favor (45 responses, 97.8%) of offering a genetic test, before diagnosis was established, to all individuals with DEE. There was no consensus when asked when they would offer genetic counseling; with responses nearly equally divided into “Prior to genetic testing” (17 responses, 44.8%) and “After genetic testing” (21 responses, 55.2%). There was no agreement between the geneticists who responded to this question.

### Neurological Assessment

#### Clinical Management

CDD is a disorder associated with DEE. Seizures often take the form of spasms, with or without hypsarrhythmia demonstrated on electroencephalogram (2, 11, 20, 31, 32), tonic seizures, and hypermotor (mixed) seizures (20). In addition, individuals may present with hypotonia (33). Male children with *CDKL5* mutations are believed to be more severely affected and have a higher frequency of epileptic (infantile) spasms and brain atrophy (34).

##### Survey

Regarding formal assessments by a pediatric neurologist, 97.6% (40 responses) of respondents felt individuals should be seen at baseline, and thereafter regularly. Asked whether individuals should be seen by a pediatric epilepsy specialist at baseline and regularly, the response was the same with 95.2% (40 responses) in agreement.

While Sudden Unexpected Death in Epilepsy (SUDEP) is reported to occur in individuals with CDD, data from large cohort studies suggests the frequency of SUDEP within the CDD population is lower than for Dravet syndrome or SCN8A-DEE, given the frequencies of these disorders (35–37). The annual risk of SUDEP among individuals with CDD remains uncertain due to the limited data.

##### Survey

Respondents were asked whether families should be informed about SUDEP at baseline. The responses were mainly in favor (86.5%, 32 responses), meeting the threshold for consensus.

##### Survey

The respondents were asked which laboratory tests should be carried out at baseline. Mixed responses included: “blood count” (18 responses, 64.3%), “vitamin D” (18 responses, 64.3%) and “urea and creatinine” (16 responses, 57.1%). Twenty-five percent (7 responses) felt no blood tests should be routinely performed. Similarly, when asked which should be carried out annually, leading responses were “blood count,” “vitamin D” (each having 16 responses, 59.3% each) and “metabolic profile with urea and creatinine” (14 responses, 51.9%). The predominance of these basic profiles and a vitamin D level suggest that the purpose of such tests is not for diagnostic benefit but to reduce the risk from associated comorbidities e.g., from gastrointestinal dysfunction or reduced mobility with associated fracture risk, as in other DEEs. There were 7 respondents who believed no annual laboratory requests should be performed (25.9%).

#### Neuroimaging

In terms of neuroimaging, there are limited, non-quantitative reports on the findings associated with CDD. One study (38) reported “cortical atrophy” in 13 of 20 girls, associated with areas of increased T2 signal in the white matter, especially in the temporal lobes in some.

##### Survey

Respondents were asked whether all individuals should have a brain MRI scan at baseline for those who have not been investigated with an MRI previously. The responses did reach a consensus with 70.3% (25 responses) responding “Yes.” As a follow-on, those who had responded “Yes” were asked whether all individuals should have a DTI brain MRI scan at baseline. Currently DTI is an area of research interest with no reports published in relation to CDD. The majority did not feel this was required; “No” (76.7%, 23 responses).

#### Electroclinical Findings and Use of Electroencephalogram

Individuals with CDD typically present with epileptic spasms within the first 4 months of life and subsequently manifest epileptic encephalopathy (32, 38, 39). Electroclinical findings in the first year of life include a peculiar seizure pattern with “prolonged” generalized tonic-clonic events, lasting 2–4 min, consisting of a tonic-vibratory contraction, followed by a clonic phase with a series of spasms, gradually transitioning into repetitive distal myoclonic jerks (40). The EEG during these seizures shows a bilateral, synchronous initial flattening, followed by repetitive sharp waves and spikes. Atypical hypsarrhythmia is often seen in infancy, developing into multifocal abnormalities in older individuals (38). Typical EEG findings develop over time and are not manifest in young infants. This likely reflects limited functional cortical organization in young infants, necessary to propagate and sustain an electrical discharge, and limited interhemispheric transmission from commissural immaturity (41, 42). Early EEG findings can vary from normal background to moderate slowing, with superimposed focal or multifocal interictal discharges and rarely, a burst-suppression pattern (40). In a follow-up of children older than 3 years, about half experienced seizure remission while continuing anti-seizure drugs, with the other half continuing to have intractable spasms, often associated with multifocal and myoclonic seizures (38, 39).

##### Survey

Most (86.0%, 37) respondents supported an EEG at baseline, regardless of clinical seizures. Most (76.9%, 30) respondents favored EEG performed to capture epileptic spasms before treatment. For less typical seizure-like events, respondents were asked whether an EEG should be repeated to capture and classify spells of unclear clinical significance. Responses were in favor, with 97.6% recommending this (40 responses). There was no consensus when asked what duration of an EEG to request. The leading response was “Routine (under 2 h)” (18 responses, 51.4%). The variation of responses may reflect the availability of prolonged EEG.

#### Seizure Management–Use of Antiseizure Drugs and Ketogenic Diet

Seizures associated with CDD typically present in early infancy, with a wide spectrum of semiologies, and are often refractory to treatment (22, 23). The most common seizure types in CDD are epileptic spasms (often without hypsarrhythmia) and tonic seizures that may cluster (20). It is uncertain what proportion of epileptic spasms are attributable to CDD, however one study identified 3 patients with pathological variants in *CDKL5* among 73 patients with epileptic spasms (43). Other seizure types have been described including atonic, atypical absence, focal with motor components, myoclonic, typical absence and tonic-clonic (44). To have pathological variants in *CDKL5* without associated seizures is extremely rare but has been reported (22) although this is unlikely to affect CDD being considered a DEE.

The treatment of epileptic spasms encompasses aspects of seizure control, side-effects and longer-term neurodevelopmental outcomes. O'Callaghan et al. performed a multicentre, open-label randomized controlled trial to investigate the effect of treatment options, either oral prednisolone (10 mg four times a day) or intramuscular tetracosactide (0.5 mg (40 IU) on alternate days), with or without oral vigabatrin (100 mg/kg per day) (45). The primary outcomes at 18 months, independently assessed, were neurodevelopmental outcomes and the frequency of seizures. While this study was not focussed on the epileptic spasms associated with CDD, it identified that earlier seizure control was a predictor of better developmental and epilepsy outcomes at 18 months. While earlier seizure control was obtained in the combination therapy group, it was surprising that there was no statistically significant difference in developmental or epilepsy outcomes at 18 months between the two groups (combination therapy or hormonal therapies alone). The authors explained this incongruity with the suggestion that those who had not responded to hormonal therapy alone would have rapidly received additional vigabatrin and therefore received combination therapy. Furthermore, any improvement in development associated with earlier cessation of seizures with combination treatment, may be undermined by the potential negative side-effects of vigabatrin such as drowsiness and visual field defects, as listed among others in the British National Formulary for Children. Studies assessing neurodevelopmental and seizure outcomes would be welcome for individuals with epileptic spasms associated with CDD, in light of reports of worse seizure outcomes with hormonal therapy for individuals with CDD. One study (22) assessed seizure variables in relation to CDD genotype and found that with a median age of questionnaire completion at 5 years, those who had previously been treated with corticosteroids had more frequent seizures than those who had never been treated, irrespective of a history of epileptic spasms.

Studies looking at the efficacy of anti-seizure drugs in the treatment of CDD-related epilepsy have frequently shown only temporary and frequently paradoxical (exacerbation) responses to various anti-seizure drugs, despite the use of medications with different mechanisms of action (23). In one study looking at the effect of anti-seizure drugs in 39 individuals with CDD (23), the highest, but still very low, responder rate after 12 months was reported with sodium valproate (9%, 3 individuals) whereas there was a very low number of individuals that responded to phenytoin, felbamate, carbamazepine and clonazepam. Drug response was defined as a more than 50% reduction in the preceding 4 weeks, compared to 4 weeks in the baseline period before starting the new anti-seizure drug. In this study, steroids/ACTH had a 19% (5) responder rate at 3 months but 0% response rate at 12 months. Similarly, vigabatrin had a 32% (8) responder rate at 3 months but just a 4% (1) responder rate at 12 months (23). For patients with earlier onset epilepsy with focal epileptiform activity, there is evidence supporting the use of sodium channel blockers, such as oxcarbazepine, carbamazepine and lacosamide (46).

Initial apparent benefit with subsequent loss of anti-seizure drug efficacy over time in the management of epilepsy associated with CDD has been described as the “honeymoon effect” (2, 22). This was first described following analysis of caregiver reports on the effects of anti-seizure medication on seizures from caregivers of 163 individuals with CDD with epilepsy registered in the CDKL5 Disorder Database (22). It was found that fewer than half (43%, 71/163) of caregivers reported ever having had more than 2 months of seizure freedom. Typically the honeymoon period had a median onset of 2 years (for 74%, 52/70) and a median duration of 6 months (for 84%, 59/70).

##### Survey

Respondents were asked to rank their first, second, third and fourth-line therapies for epileptic spasms associated with CDD. There was no consensus for any of the first, second, third or fourth line suggested therapies, although the standard treatments of vigabatrin, steroids and the combination of these featured most strongly. For first line therapy, 37.5% (15 responses) favored combination therapy (steroids and vigabatrin), 35% (14 responses) favored steroids alone and 27.5% (11 responses) favored vigabatrin alone. No responder suggested use of ketogenic diet as a first line therapeutic option. Similarly, there was no consensus among second line therapy options, however among a choice of steroids, vigabatrin, combination of these or the ketogenic diet, the ketogenic diet was selected by nearly a quarter (23.1%, 9 responses) as a second line therapeutic option. The ketogenic diet similarly made up an increasing preference (17 (54.8%) and 10 (41.7%) responses) for third and fourth line therapy preferences. The ketogenic diet was considered by respondents as early in the management of seizures as a second or third line therapy option, with few other epilepsies, e.g., *SLC2A1* mutation (47), prompting such early consideration.

Lim et al. (48) studied the use of the ketogenic diet to manage refractory epilepsy associated with CDD. They found that of the approximately half of individuals with CDD who have tried the ketogenic diet, some 59% of individuals experienced improvement in seizure frequency, duration, or intensity. However, none of the individuals on the ketogenic diet became seizure-free. This lack of complete resolution of seizures, along with side-effects of the diet, led to poor long-term adherence (median duration 17 months). In a study on quality of life domains for individuals with CDD, 20% (5 of the 25 surveyed) were currently on a ketogenic diet (49).

##### Survey

The respondents were asked whether individuals should be treated with a ketogenic diet as soon as they fail their first line treatment for epileptic spasms. The responses were mixed with most in favor (23 responses, 53.5%). This response may be interpreted as encouragement for starting a ketogenic diet at the soonest moment that a first line therapy has proven inadequate for controlling epileptic spasms and that the diet may be in addition to a second line medication option (differentiating this nuance from the preceding survey responses).

While several studies looking at the use of CBD for the treatment of drug-resistant epilepsy have shown promising results, few have provided specific results for the performance of CBD in the CDD subpopulation (50). Devinsky et al. (51) undertook an open-label study exploring the use of CBD in individuals with severe, treatment-resistant, childhood-onset epilepsy including CDD, among other disorders. In individuals with CDD, the median monthly convulsive seizure frequency decreased from baseline (66.4 [*n* = 17], IQR: 25.9-212.0 to week 12 (35.8 [*n* = 11], IQR: 8.9-141.6) which was found to be statistically significant (*p* = 0.032). Further placebo-controlled randomized trials in a larger population sample are necessary to formally assess the safety and efficacy of cannabis-based products in CDD.

##### Survey

There was consensus on whether CBD (Epidiolex) should be offered for epilepsy in CDD. The responses provided strong support for this option with 92.6% (25 responses) in favor with 7.4% against (2 responses). This reflects an increasingly positive view of CBD for medicinal uses, including in the pursuit of reducing seizure burden among populations of children with mixed etiologies of drug-resistant epilepsy (52, 53).

Ganaxalone is a synthetic methyl derivative of allopregnanolone, a neurosteroid, which acts as a high-affinity allosteric modulator of GABA_A_ receptors. Ganaxalone has been trialed for epilepsies including epileptic spasms, status epilepticus and protocadherin 19 related epilepsy (2). The Marigold Study (NCT03572933) is the first Phase 3, randomized, placebo-controlled trial that evaluated adjunctive ganaxolone in patients with refractory epilepsy associated with CDD. Patients on ganaxolone experienced a median of 30.7% reduction in major motor seizure frequency compared to a 6.9% reduction in the placebo group during the treatment period relative to baseline (*p* = 0.0036, Wilcoxon Rank-Sum Test). Ganaxolone demonstrated improving trends but did not achieve statistical significance in the key secondary endpoints. Adverse events occurred in 86% of ganaxolone patients and 88% of placebo patients. Ganaxolone was generally well-tolerated with a <5% discontinuation rate in the treatment arm, with somnolence being the most frequent adverse event (36% of patients) (54).

##### Survey

Respondents were asked whether Ganaxolone should be offered, if available (dependent on regulatory approval). The unanimous response was “Yes” (27 responses, 100%), meeting the threshold for consensus. The FDA has just approved ganaxolone (Ztalmy; Marinus Pharmaceuticals) for the treatment of seizures associated with CDD, in patients aged 2 years and older.

#### Epilepsy Surgery

The effects of vagus nerve stimulation (VNS) for the treatment of refractory epilepsy for CDD has been studied (55). Of 222 patients with *CDKL5* variants where there was adequate information, 38, the equivalent of 1/6 or 17% had previous or current use of VNS. Improvement in seizure control was reported in 69% (25/36) and of them, this related to improvements in frequency in 68% (17/25), duration in 72% (18/25) or intensity in 60% (15/25). No patient with a VNS became seizure-free and termination of VNS occurred in 1 in 10 cases.

##### Survey

Respondents were asked whether individuals should be considered for VNS insertion if seizures are refractory to medications. The responses were mainly in favor (89.7%, 35 responses).

Patients with non-resectable, drug-resistant seizures with spread between hemispheres, i.e., generalization, may be considered for corpus callosotomy. In a meta-analysis of the effects of corpus callosotomy in epilepsy surgery, analyzing the impact of corpus callosotomy on 1,742 children and adults from 58 studies, it has been shown to be associated with drop attack freedom in 55.3% and complete seizure freedom in 18.8% (56). For those achieving complete seizure freedom, this favored patients whose etiology included infantile spasms (OR 3.86, 95%. CI 1.13-13.23), normal MRI (OR 4.63, 95%. CI 1.75-12.25), and a shorter epilepsy duration of <15 years (OR 2.57, 95%. CI 1.23-5.38). Interestingly, neither the presence of lateralising EEG abnormalities nor the selection of complete vs. partial corpus callosotomy made a significant impact on the outcome, unlike in the analysis of patients with drop attacks where these were associated with improved outcomes.

##### Survey

Respondents were asked whether individuals should be considered for corpus callosotomy if seizures were refractory to medications. The leading response was 71.0% in favor (22 responses), meeting the threshold for consensus.

#### Stereotypes and Movement Disorders

Hand stereotypies are reported in 80% of individuals and can negatively affect functional hand movements in 59% of females and 12.5% of males with CDD (1). Olson and colleagues (unpublished) describe self-stimulatory hand movement syndrome and repetitive leg crossing in CDD patients. Unquantified episodes of persistent, occasionally severe, choreoathetosis, akathisia, dystonia and parkinsonian features have been reported, potentially having been unmasked during temporary periods of improved seizure control or potentially secondary to polytherapy with antiseizure drugs (2).

##### Survey

Respondents were asked whether individuals should be screened for movement disorders at baseline. The responses were: “Yes” (39 responses, 100%), achieving consensus. The respondents were also asked whether individuals should be screened for movement disorders at regular clinical appointments, annually, with 100% in favor (38 responses). Respondents were 100% in favor with regard to movement disorders being treated if causing problems. Asked what would be the most suitable option, the leading responses were: “Gabapentin” (15 responses, 62.5%), “Clonidine” (13 responses, 54.2%) and “Benzodiazepines” (10 responses, 41.7%).

#### International Registry

With increased attention on therapies for CDD, prospective, randomized, and double-blind clinical trials are considered essential to establish statistical significance and thus will necessitate international collaboration (57).

##### Survey

When asked whether individuals should be offered to be enrolled in an international registry or other research studies, 100% were in favor (46 responses).

#### Neuropsychological Assessment

##### Survey

When asked whether individuals should have a neuropsychology assessment at baseline (where the diagnosis has already been made), there were mixed responses with 59.4% in favor (19 responses). Similarly, when asked whether individuals should have a neuropsychology assessment regularly, responses were: “Yes” (26 responses, 68.4%). This did not meet the threshold for a consensus of opinion.

#### Somnology

Sleep-related difficulties are reported in over 85% of individuals with CDD, sometimes dubbed “all night parties” with problematic night-waking reported in up to 58.5% (1, 2, 19) and males more severely affected (19). Sleep apnoeas have been documented in both individuals and mouse models of CDD (58, 59). The odds of reported sleep difficulties was higher in the 5–10 year age group than the under 5 year group (19).

##### Survey

Respondents in our survey were asked whether individuals should have their sleep assessed at baseline. The leading response met the threshold for consensus with 92.3% (36 responses) in favor. Similarly, there was consensus when respondents were asked whether individuals should have their sleep assessed annually with 85.7% (30 responses) in favor. When respondents were asked which drug or drugs could be used to help with sleep, the leading response, “Melatonin” (35 responses, 53.8%), did not meet the threshold for consensus of recommended first choice, however, was more popular than the second most selected answer, “Clonidine” (16 responses, 24.6).

### Therapy Assessments and Interventions

#### Neuro-Rehabilitation Assessment

Neuro-rehabilitation services, sometimes referred to neuro-developmental or neuro-disability services, are part of the care of individuals with CDD. Assessing function and response to therapies is important in guiding and interpreting the findings of future research into therapies for CDD (60). A collaborative professional and caregiver-based standardized assessment method was designed using four cycles of a Delphi process, the CDD Clinical Severity Assessment (CCSA). This involved clinicians from the International Foundation for CDKL5 Research Centers of Excellence (COE) consortium and the National Institutes of Health' Rett Syndrome, MECP2 Duplication Disorder, and Rett- Related Disorders Natural History study consortium (U54 HD061222; ClinicalTrials.gov: NCT00299312/ NCT02738281). Initial consensus was provided by clinicians, researchers, industry, patient advisory groups and the parents of a child. The CCSA reviewed 53 items, 27 reported by parents and 26 reported by clinicians. It has recently been developed (61) and validated to enable its implementation for the assessment of outcome measures, as per FDA requirements (62, 63).

The final CCSA will be 50% clinician assessment of motor, cognition, behavior, vision, speech and autonomic function domains. The other 50% will be parent-led assessment, complimentary to the design and structure of the clinician assessment. The aims of the CCSA are to support design and interpretation of research, evidence-based management choices in CDD and identification of current patient needs. Specific items capture levels of functioning in the gross motor, hand function, communication and behavior domains.

##### Survey

We asked whether individuals should be offered a referral to a neuro-rehabilitation service at baseline, to assess equipment needs and diagnose or improve problems with mobility and hand function and to prevent contractures. There was strong support for this with 91.9% of respondents (34 responses) in favor. Similarly, when asked whether individuals should be offered a referral to a neurorehabilitation service annually for the same purpose, 92.1% (35 responses) were in favor.

#### Development Assessments

CDD is associated with global developmental delay including intellectual disability. Most individuals are severely impaired. In one study (18), data for 108 females and 16 males, registered with the International CDKL5 Disorder Database, were collected. Over half of females could sit on the floor and nearly a quarter could walk 10 steps. Most females and few males were able to pick up a large object. Those with a late truncating variant displayed better levels of ability than those with no functional protein. Subsequent research has expanded the correlations of the genotype-phenotype (20).

This work was also performed using an expanded cohort from the same International CDKL5 Disorder Database (24). The study looked at genotype-phenotype findings for 385 individuals with CDD. They then assessed genotype-phenotype relationships for 13 recurrent *CDKL5* variants and compared these with previously analyzed historic variant groups. Developmental scores and severity assessments were performed using the CDKL5 Developmental Score (CDS) and an adapted CDKL5 Clinical Severity Assessment (CCSA). Individuals with the missense variant, p.Arg178Trp, had the highest mean adapted CCSA and lowest mean developmental scores. They also found that p.Arg559^*^ and p.Arg178Gln produced severed phenotypes whereas p.Arg134^*^, pArg550^*^ and p.Glu55Argfs^*^20 produced milder phenotypes. This study identified trends between variants and phenotypes and updated historic genotype-phenotype reports.

Regression, if encountered, is often related to worsening of seizure control and the presumed effect of epileptic encephalopathy (1, 18, 32, 33, 64). In girls, walking is attained by 22%, raking grasp by 49% by 5 years and pincer grasp by only 13% at any point (18, 65).

##### Survey

We asked whether individuals with CDD should have developmental assessments and 100% were in favor (44 responses), with 75% (24 responses) proposing, “Soon after diagnosis,” meeting the threshold for consensus. Nearly all (95.3%, 41 responses) of respondents felt developmental status assessment should be repeated. Nearly all (92.3%, 36 responses) felt the assessments ought to occur at key developmental points and periods of transition, proposed as during infancy (0–3 years), preschool age (3–6 years), pre-middle school age (6–9 years), adolescent age (12–16 years, early adulthood (18–25 years) and as needed thereafter.

#### Ophthalmology

CDD is associated with cortical visual impairment (CVI) with approximately 75% having cortical visual impairment (20).

##### Survey

The respondents were asked whether individuals should have a detailed vision assessment at baseline. The responses were: “Yes” (38 responses, 100%). Similarly, respondents felt individuals should have an annual vision assessment with all in favor (29 responses, 100%). When asked whether individuals with CDD should be referred to an ophthalmology specialist familiar with cortical visual impairment, for assessment, the responses were strongly (100%, 37 responses) in favor. For management by an ophthalmology specialist familiar with CVI, the responses were also 97.1% (34 responses) in favor.

#### Speech and Language Assessment and Communication Aids

As part of global developmental delay and associated cortical visual impairment, individuals with CDD experience difficulties with communication (18). In one study (65), it was found that under half of individuals could babble by the age of six (43/97, 44%) and under a quarter could say single words by the age of seven (17/105, 16%). Only 7.5% of females achieve speaking in full sentences (18) with males 80% less likely than females to be able to use advanced communication methods (OR 0.17, 95% CI 0.04–0.71). Upon assessment and categorization of highest communication ability, it was found that 26% were able to use spoken language, sign language and abstract symbols, followed by 39% who were able to use complex gestures, vocalizations and concrete symbols with 33% able to use only simple communication alone (such as body language, early sounds, facial expressions and simple gestures). While speech difficulties can present with other features suggestive of autism, this diagnosis is infrequently made while in the context of severe global developmental delay (2).

There have been few studies published reviewing the use of non-verbal communication aids for individuals with CDD. Unpublished data by Olson et al., reviewed the use of devices such as switches and eye gaze technology-based communication aids. They found that in those unaffected or mildly affected by cortical visual impairment, such devices provided assistance for some with CDD. A recent systematic review has investigated outcomes and uptake barriers for the pediatric population with complex disabilities using eye gaze assistive technology (66). This analysis reviewed the use of eye gaze technology on the World Health Organisation's International Classification of Functioning, Disability and Health Framework. There were 11 articles suitable for review, of which eight assessed communication and of which six reported enhanced communication outcomes. The review highlighted poor methodological quality and/or low level evidence, limiting the review's findings and reflecting a need for further published and high-quality evidence.

##### Survey

When asked whether individuals with CDD should be checked and assessed for augmentative and assistive communication aids such as switches, touch pads or eye gaze aids, respondents were unanimously in favor (41 responses, 100%).

#### Orthopedic, Physiotherapy and Occupational Therapy Assessments

Orthopedic concerns are a potential consequence of hypotonia and can lead to scoliosis, with 68.5% of individuals affected by 10 years (1, 19).

##### Survey

Asked whether individuals should have a hip and spine X-ray, most responses were: “If there is a clinical concern” (31 responses, 77.5%), reaching the threshold required for consensus. Respondents did not favor individuals with CDD having a routine orthopedic (specialist surgeon) review at baseline, with the leading response being not in favor (22 responses, 73.3%). Equally, when asked whether individuals should have a routine yearly orthopedic review, the responses leaned toward not being in favor (15 responses, 53.6%). Whether reflecting concerns (e.g., pertaining to reduced mobility or a ketogenic diet) when asked whether individuals with CDD should be offered a screening test for osteopenia (such as wrist X-ray or DEXA scan), the leading responses was: “If clinically indicated” (28 responses, 82.4%).

Consensus guidelines for the approach to screening and management of scoliosis and osteopenia are not available for CDD however a consensus of routine management for optimal bone health in Rett syndrome has been developed and is likely relevant to individuals with CDD until higher level evidence becomes available (25, 67–69).

Fu et al. provided observational data for 913 females with classic Rett Syndrome. They identified that severe scoliosis was found in 251 participants (27%), 113 of whom developed severe scoliosis during follow-up assessments with 168 (18%) having surgical correction. The study proposed the implementation of spinal bracing when spinal curvature reaches 25°, in the hope of retarding or minimizing further progression. Beyond 40°, the authors strongly promoted surgical intervention. Each study suggests annual evaluations for both of these issues along with guidelines for management and referrals.

##### Survey

There was consensus in favor when asked individuals with CDD should be offered Physical Therapy (PT) assessment at baseline (where diagnosis has already been made) with 97.8% of respondents in favor (44 responses). Equally 97.8% (44 responses) felt that individuals with CDD should have access to PT regularly for ongoing issues.

##### Survey

Asked whether individuals should be offered an occupational therapy (OT) assessment at baseline (where diagnosis has already been made), the responses strongly in favor (38 responses, 92.7%). Similarly, when asked whether individuals with CDD should have access to OT regularly for ongoing issues, the responses were strongly in favor (42 responses, 100%).

#### Educational

Individuals with CDD face difficulties such as communication difficulties and cortical visual impairment. Interventions, such as visual attention tracker, may assist in informing the wider team whether educational interventions are providing benefit (70).

##### Survey

Asked whether educational accommodations for visual impairment should be provided, 97.6% (41 responses) were in favor. More broadly, 92.1% (35 responses) were in favor when asked whether educational support provided in formal educational plans should be reviewed at baseline. Similarly, respondents felt a review of these should be performed annually, with 94.9% (37 responses) in favor.

### Systemic

#### Auxology

Five individuals with CDD were reported to have normal head circumferences at birth and over the subsequent 2 years develop postnatal microcephaly (64). Similarly, deceleration of head growth has been described in 11 out of 20 (55%) individuals with CDD (33). Microcephaly has been associated with an increased degree of functional impairment (71).

##### Survey

When asked whether head circumference, weight, height should be each checked at baseline, respondents were in favor; 100% (46 responses), 97.8% (45 responses) and 97.6% (42 responses), respectively. Similarly, when asked whether height and weight should be checked annually, 100% (43 responses) were in favor.

#### Gastrointestinal Management Including Assessment and Management of Feeding

Patients with CDD may experience dysphagia and require gastrostomy (2). Evidence suggests that gastrostomy tube feeding for pediatric patients with neurological impairments may reduce the risk of death although associated with an increased the risk of severe pneumonia (72). Guidelines produced by the European Society for Pediatric Gastroenterology, Hepatology and Nutrition, for the evaluation and treatment of gastrointestinal and nutritional complications in children with neurological impairment, recommends the use of enteral tube feeding in cases of unsafe of inefficient oral feeding, preferably before the development of undernutrition, and that a gastrostomy is the preferred way to provide intragastric access for long-term tube feeding for this population. Aside from nutritional difficulties affecting growth, a gastrostomy tube may improve caregiver quality of life, assist in the administration of fluids and/or a ketogenic diet and, through compliance with medications and/or ketogenic diet, may reduce seizure burden (73, 74). A review of patients from the CDKL5 Disorder Database found that 20.7% of individuals were fed exclusively by gastrostomy or nasogastric tube (19) but this prevalence may be as high as 43% among individuals with CDD, following analysis of patients based in the United States of America (75) (154 individuals identified from data held by Centers of Excellence and 40 identified from the NIH's Natural History of Rett and Related Disorders database). In a smaller study on quality of life domains for those with CDD, as many as 56% (14/25 surveyed from the CDKL5 international registry) had a gastrostomy (49).

##### Survey

Respondents were asked whether gastrointestinal complications such as constipation, air swallowing and acid reflux should be assessed at each clinic visit annually. The responses were strongly in favor (43 responses, 97.7%). Asked whether individuals should be referred to a Gastrointestinal specialist, responses were in favor (92.0%, 23 responses). When asked whether individuals should be referred to a Nutrition specialist, responses were also in favor (30 responses, 96.8%). When asked when swallowing coordination should be formally assessed (i.e., by Speech and Language Specialists) most felt this should be, “Only if there are concerns” (25 responses, 61.0%). Respondents were more strongly in favor of individuals being offered an informal speech therapy assessment at baseline (where diagnosis has already been made) (38 responses, 92.7%). Similarly, a large majority felt that non-specialist feeding, and swallowing should be assessed at annual clinical reviews (36 responses, 90.0%). Respondents were asked when a gastrostomy should be considered, with responses meeting consensus in the selection of, Either (including, “When weight or BMI inappropriately plateaus or tails” or “When swallowing is considered unsafe”) (31 respondents, 72.1%). A third of respondents (14 responses, 32.6%) felt this should be limited to “When swallowing is considered unsafe”.

#### Respiratory Assessment

Breathing abnormalities with CDD have been reported and include hyperventilation in 13.6%, breath holding in 26.4% and aspiration in 22.6% (19). The respondents were asked whether a formal respiratory review should be offered routinely at baseline, including a sleep study, to all individuals. There was no consensus however the lead response was “Only if clinically indicated” (28 responses, 66.7%). Similarly, when asked whether individuals should be referred to a pulmonologist/respiratory clinician, 81.0% reported “Only if clinically indicated” (34 responses). However, when respondents were asked whether a non-specialist assessment for breathing disorders, including hyperventilation, breath-holding and other conditions should be offered at each clinic visit annually, the leading response met the threshold for consensus with 90.5% (38 responses) in favor.

#### Cardiovascular Assessment

Parents of children with CDD may have concerns about the risk of cardiac arrhythmias and, in one caregiver survey, arrhythmia was reported in 11 out of 29 individuals with CDD who had been investigated with electrocardiogram (ECG) (76). Despite parental reports of arrhythmias, there is a lack of data on the rates of arrhythmia among individuals with CDD [from published reviews based on a cohort of 93 individuals published from the International Foundation for CDKL5's Research Centers of Excellence (2)].

##### Survey

When asked whether individuals should be routinely screened for cardiac issues at baseline (where the diagnosis has already been made), the most common responses were: “Yes” (26 responses, 78.8%), meeting the threshold for consensus. Similarly, when asked whether individuals should have an ECG at baseline (where the diagnosis has already been made) the most cited response was “Yes” (31 responses, 86.1%) achieving consensus. However, there was a lack of consensus when respondents were asked whether individuals should have a routine annual ECG, the leading responses were “Yes” (19 responses, 63.3%). Equally, when respondents were asked whether the individuals should have an echocardiogram at baseline (where the diagnosis has already been made), the leading responses were: “No” (15 responses, 57.7%) with fewer in favor of this (11 responses, 42.3%). Furthermore, when respondents were asked whether individuals should have a routine annual echocardiogram, leading responses were: “No” (23 responses, 88.5%). Lastly, when asked whether individuals should have a routine annual cardiological review by a cardiology specialist, the leading response was “No” (17 responses, 73.9%).

#### Dermatology

##### Survey

The respondents were asked whether individuals should have a routine check for pressure ulcers and skin breakdown at baseline (where the diagnosis has already been made). The lead response was in favor (38 responses, 90.5%). Asked whether individuals should have a regular skin check at their annual clinic review, responses were similarly in favor (38 responses, 95%).

#### Urinary Tract Care

##### Survey

When respondents were asked whether bladder-related issues should be checked regularly (e.g., urinary retention and urinary tract infections), it was felt this was appropriate with 94.1% of respondents in favor (32 responses).

#### Audiological

##### Survey

All survey respondents were in favor of individuals with CDD having an audiological assessment in the form of Automated Auditory Brainstem Response (AABR) screening (100%, 36 responses).

#### Dental Care

##### Survey

All survey respondents were in favor that individuals should have baseline and regular dental checks upon diagnosis of CDD (100%, 40 responses).

### Financial

#### Survey

Respondents were asked whether financial support options should be explored as a baseline assessment upon diagnosis of CDD and annually, during clinic reviews. The responses were 100% with 43 responses and 39 responses respectively, both in favor.

### Summary of Areas Not Meeting Threshold for Consensus

While there was no consensus in the current study regarding the timing of genetic counseling, the ACMG has provided recommendations for genetic counseling prior to and following genetic testing (77).

Notably, for a condition predominantly regarded as an epileptic encephalopathy in the domain of epilepsy management, there was no consensus on the first, second or third line choices of anti-seizure drug. This may reflect varying clinician preferences or clinicians individually tailoring management to meet the specific needs and varying seizure types of their patients. Nevertheless, vigabatrin, steroids and the combination of these featured most strongly, favoring combination therapy as first line (37.5%, 15 responses) for the management of epileptic spasms.

### Summary of Areas Meeting Threshold for Consensus

The following table (Table 1) outlines the responses in the survey which met the pre-defined 70% requirement for consensus status, and their recommended timepoints (“baseline,” “annually” or “if clinically indicated”).

**Table 1:** Recommendations for the management of individuals with CDD with suggested timepoints for completion.

	**Baseline**	**Annually**	**If clinically indicated**
**Genetic testing**	Genetic testing should be offered to all individuals with DEE to confirm diagnosis.		
**Neurological**			
Clinical management	Review by a pediatric neurologist and (if not the same professional) an epilepsy specialist. Families should be informed about Sudden Unexpected Death in Epilepsy.	Review by a pediatric neurologist and (if not the same professional) an epilepsy specialist.	
Neuroimaging	Individuals should be investigated with a brain MRI scan.		
EEG	EEG (regardless of clinical seizure status).		An EEG should be repeated to capture and classify spells of unclear clinical significance.
Anti-seizure drugs			Individuals with seizures should be offered Ganaxolone, if available. Equally, CBD (Epidiolex) should be offered for epilepsy with CDD, provided this met legal and regulatory requirements.
Epilepsy surgery	Individuals should be considered for a VNS insertion if seizures are refractory to medications. Individuals should be considered for corpus callosotomy if seizures are refractory to medication.		
Stereotypes and movement disorders	Individuals should be screened for movement disorders and have these treated if causing problems.	Individuals should be screened for movement disorders and have these treated if causing problems.	
International registry	All individuals with CDD should be offered to be enrolled in an international registry of other research studies		
Somnology	Individuals should have their sleep assessed by their clinician.	Individuals should have their sleep assessed by their clinician.	
**Therapy assessments and interventions**			
Neurorehabilitation	Referral to a neuro-rehabilitation service to assess equipment needs and diagnose problems causing impairment of mobility or hand function and to prevent contractures.	Referral to a neuro-rehabilitation service to assess equipment needs and diagnose problems causing impairment of mobility or hand function and to prevent contractures.	
Development			Development should be assessed during infancy (0–3 years), preschool age (3–6 years), pre-middle school age (6–9 years), adolescence age (12–16 years, early adulthood (18–25 years) and as needed thereafter.
Ophthalmology	Individuals should have a detailed vision assessment. Individuals should be referred for assessment and management of cortical visual impairment by an ophthalmologist familiar with this condition.		
Communication	Individuals should be offered a speech therapy assessment and assessed for augmentative and assistive communication aids such as switches, touch pads or eye gaze aids.		
Orthopedics			Hip and spine X-ray if there is a clinical concern. Screening test for osteopenia (such as wrist X-ray or DEXA scan) if there is a clinical concern
Physiotherapy (PT)	Individuals should be offered PT assessment.		Access to PT regularly for any ongoing issues.
Occupational therapy (OT)	Individuals should be offered an OT assessment.		Access to OT for any ongoing issues.
Educational	Formal educational plans should be reviewed.	Formal educational plans should be reviewed.	Educational accommodations should be made if visual impairment is present.
**Systemic**			
Auxology	Assessment of head circumference, weight and height.	Assessment of head circumference, weight and height.	Assessment of head circumference, weight and height.
Gastrointestinal management including assessment and management of feeding	Assessment of gastrointestinal complications such as constipation, air swallowing and acid reflux. Individuals should be referred to a Gastrointestinal specialist as well as a Nutrition specialist. Non-specialist feeding and swallowing should be assessed during clinic reviews.	Assessment of gastrointestinal complications such as constipation, air swallowing and acid reflux. Non-specialist feeding and swallowing should be assessed during clinic reviews.	A gastrostomy should be considered either when weight plateaus or BMI tails inappropriately or when swallowing is considered unsafe.
Respiratory	A non-specialist respiratory assessment to screen for breathing disorders, including hyperventilation, breath-holding or other conditions.	A non-specialist respiratory assessment to screen for breathing disorders, including hyperventilation, breath-holding or other conditions.	Referral to a pulmonologist/respiratory clinician.
Cardiology	Screening for cardiac issues and this should include an ECG.		
Dermatology	Individuals should have a routine skin check for pressure ulcers and skin breakdown.	Individuals should have a routine skin check for pressure ulcers and skin breakdown.	
Urology	Bladder related issues should be checked regularly (e.g., to assess for urinary retention and urinary tract infections)	Bladder related issues should be checked regularly (e.g., to assess for urinary retention and urinary tract infections).	
Audiology	Individuals should have an audiological assessment in the form of auditory brainstem response (AABR) screening.		
Dental care	Individuals should have a dental check	Individuals should have a dental check.	
Financial	Financial support options should be explored.	Financial support options should be explored.	

There were many areas of consensus recommendations identified. The majority of these are for completion at baseline. There is an emphasis upon holistic care, such as the monitoring of systemic functions and educational needs, with certain areas recommended to be reviewed, not only at baseline, but also annually and if clinically indicated. These included the monitoring of growth, the need for a regular review of feeding and swallowing, and non-specialist screening for respiratory difficulties.

A comprehensive neurological assessment is encouraged at baseline. The consensus recommendations are for the individual with CDD to be reviewed by a pediatric neurologist with experience in managing epilepsy, clinician discussion to inform families about the risk of SUDEP, completion of a baseline MRI and EEG, consideration for epilepsy surgery, screening for the presence of a movement disorder, registration with the CDKL5 international registry and a review of the individual's sleep. Despite limited published evidence on the use of novel antiseizure drugs for CDD in the literature, Ganaxolone and Epidiolex are encouraged to be offered for epilepsy associated with CDD, if clinically indicated, dependent on FDA and EMA approvals and legal and regulatory requirements, respectively.

## Discussion

CDD is a debilitating condition where there is an urgent need for further development of management options. To achieve these necessary advances will require large scale and international, collaborative efforts to evaluate potentially effective interventions in sufficiently powered clinical trials. Progress will rely heavily on cooperation between international medical and scientific professionals, affected families, industry and funding organizations (57). The extensive experience of the author group includes those with direct experience in CDD management including authors of a clinically relevant CDD severity assessment tool (78). We hope that this survey adds to the current knowledge base concerning clinical aspects of care and provides a useful proposed standard of care elucidated by the agreed areas of consensus. These recommendations can support clinicians with less experience of CDD and act as a catalyst for further research that would aim to increase capacity for evidence-based management in CDD.

## Limitations of Survey

In the survey there were occasions when incomplete responses were obtained, ie. fewer than 47 responses per question. This could represent difficulties in selecting the options available (for example, when no “other” option for selecting preferred first-, second- or third-line antiseizure drug preferences) or technical difficulties with the online survey.

For answers where respondents did not have experience in this area, answering “I am not qualified to answer” or “I do not know,” responses were excluded from analysis which led to a reduced number of responses included in the analysis. This was notable for certain technical questions, such as whether an MRI with DTI should be performed at baseline (8 respondents selected “Do not know/Do not feel strongly” and 6 selected “I am not qualified to answer”) and also for evolving areas of research interest, such as whether CBD (Epidiolex) should be offered for epilepsy in patients with CDD, where 6 respondents selected “Do not know/Do not feel strongly” and 7 selected “I am not qualified to answer.”

Certain answers provided professional discretion and may have been subject to personal interpretation, for example, in the use of screening tests for osteopenia, the leading response was ‘If clinically indicated' however the indications (e.g., poor mobility, fracture, poor height velocity, bony malformations) in this and other situations were not directly specified.

We invited respondents to provide additional feedback on areas of CDD management that were not covered in the survey. While the survey was designed and constructed with broad support at the outset, we acknowledge that some detail may have been overlooked and therefore we invited comments and suggestions for any missed areas at the end of the survey. There were few responses (4 out of 47) possibly suggesting the survey was felt to be sufficient by the majority. Of the responses, the feedback included a need to explore access to support groups and the contacting of other families. Another responder questioned whether mosaicism should be discussed within genetic counseling. This response may be in reference to reported findings of somatic mosaicism in patients with CDD (79, 80) or germline mosaicism with *CDKL5* which was described in one family with two daughters with CDD found to have the same *CDKL5* variant (c.283-3_290del) with parents that tested negative for *CDKL5* variants in all tissues (81).

Further comments included reference to gynecological needs, not described in the survey. The responder queried whether clinicians should consider screening for precocious puberty or referring to gynecology, in the event of problems with menses. This suggestion addresses the unaddressed gynecological facet of CDD holistic care but may also be in reference to precocious puberty which has been described with CDD (82).

Reflective of increasing literature on CDD, one of the respondents suggested whether individuals should have an “anticipatory care plan” and whether this should be reviewed at least annually. This countered another piece of feedback: a concern that being too prescriptive with a potentially “exhaustive” list of management recommendations could heighten parental anxiety (if they feel they or those looking after their child are not fulfilling it). Clinicians managing CDD may need to decide whether to be “anticipatory” or, conversely, more “problem-driven” and which approach may be more appropriate for the individual and their family.

As with other work aiming to bring consensus to the understanding and management of CDD, our project lacks an objective “gold standard,” instead being designed with the topics and subtopic questions selected through limited published data, Delphi consensus and expert opinion. In the absence of a high level of evidence, Delphi consensus is considered the best available guidance. We recognize that despite our collective experiences, we are each limited by these experiences and the field still has much to learn regarding the breadth of patient experiences, potential treatments and outcomes. The concept of an “expert” is quite relative with regards to rare disorders such as CDD.

Given these shortcomings, additional discussion and study is needed regarding several issues. While our panel was equivocal, ACMG guidelines that genetic counseling should be provided at all phases of genetic testing (77) seems most prudent. Similarly, an approach toward scoliosis and osteopenia similar to that proposed for Rett Syndrome (25, 67–69) should be provided. All treatments carry risk of potentially significant morbidity and mortality that should be carefully reviewed with families so that informed treatment decisions should be made. Addressing a complete algorithm for use of anti-seizure medications, including variations with age and seizures types, was beyond the scope of our approach, but should be considered as a completely separate effort. A standard approach to epilepsy management in CDD including avoidance of polypharmacy should be considered, even though the literature indicates significant medical resistance (22).

Consistent with this, our survey indicates that medication and surgical options that may be offered to other individuals with medically resistant epilepsy, due to other causes, should also be offered to individuals with CDD. There has not been strong evidence until recently to support any specific treatment interventions in this population including steroids, surgery or any other specific anti-seizure medications. However, following the large international placebo controlled trial of ganaxolone, the FDA has just approved ganaxolone (Ztalmy; Marinus Pharmaceuticals) for the treatment of seizures associated with CDD, in patients aged 2 years and older.

Families should be part of the decision-making process and presented with both the clinician's experience and that of the broader community and literature. Our approach has been that management in rare diseases should be a “team sport.” This study was prompted by frequent emails to each other to discuss potential approaches to increase our collective pool of experience; the community is encouraged to join us.

## Data Availability Statement

The original contributions presented in the study are included in the article/supplementary material, further inquiries can be directed to the corresponding author.

## Author Contributions

All authors listed have made a substantial, direct, and intellectual contribution to the work and approved it for publication.

## Funding

HO was supported by NINDS K23 NS107646-04, PI Olson. TB was supported by the Ponzio Family Chair in Neurology Research to the Children's Hospital Colorado Foundation.

## Conflict of Interest

JD Consultancy for Marinus, Ultragenyx, Avexis, Anavex, and Newron; any remuneration went to Telethon Kids Institute. MM works as a pediatric researcher with investigator initiated studies funded through industry (PTC Therapeutics). EP is on the advisory board of Marinus Pharmaceuticals and has consulted for Biomarin Pharmaceuticals and Zogenix. JC has acted as an investigator for studies with GW Pharma, Zogenix, Vitaflo, Ovid, Marinius and Stoke Therapeutics. She has been a speaker and on advisory boards for GW Pharma, Biocodex, Zogenix, and Nutricia; all remuneration has been paid to her department. Her research is supported by the National Institute of Health Research (NIHR) Biomedical Research Centre at Great Ormond Street Hospital. She holds as endowed chair at UCL Great Ormond Street Institute of Child Health; she holds grants from NIHR, EPSRC, GOSH Charity, ERUK, the Waterloo Foundation and the Great Ormond Street Hospital Biomedical Research Centre. SA has received funding from GW Pharmaceuticals, Norvartis, PTC Therapeutics, Boston Scientific, Nutricia, UCB, BioMarin, LivaNova, Medtronic, Desitin, Ipsen, CDKL5 UK, TSA and the National Institute for Health Research. HO received consulting fees from Takeda Pharmaceuticals and Zogenix regarding clinical trial design, Ovid Therapeutics regarding clinical trial results, Marinus Pharmaceuticals regarding CDKL5 Deficiency Disorder, and has done consulting for the FOXG1 Research Foundation. TB performed consultancy for Ovid, GW Pharmaceuticals, International Rett Syndrome Foundation, Takeda, Neurogene, Ultragenyx, Zogenix, GrinTherapeutics, Alcyone, Acadia, Neuren and Marinus; Clinical Trials with Acadia, Ovid, GW Pharmaceuticals, Marinus and RSRT; all remuneration has been made to his department. The remaining authors declare that the research was conducted in the absence of any commercial or financial relationships that could be construed as a potential conflict of interest.

## Publisher's Note

All claims expressed in this article are solely those of the authors and do not necessarily represent those of their affiliated organizations, or those of the publisher, the editors and the reviewers. Any product that may be evaluated in this article, or claim that may be made by its manufacturer, is not guaranteed or endorsed by the publisher.

## Abbreviations

CBD: Cannabidiol
CDD: CDKL5 Deficiency Disorder
CDKL5: Cyclin Dependent Kinase-like 5
DEE: Developmental Epileptic Encephalopathy
DEXA: Dual-Energy X-ray Absorptiometry
DTI: Diffusion Tension Imaging
ECG: Electrocardiogram
EEG: Electroencephalogram
MRI: Magnetic Resonance Imaging
SUDEP: Sudden Unexpected Death in Epilepsy.
